# Sialolithiasis: An Unusually Large Submandibular Salivary Stone

**DOI:** 10.7759/cureus.41859

**Published:** 2023-07-13

**Authors:** Gargi Jadaun, Deepa Pillai, Tejal Ragji, Saeeda Kharodia

**Affiliations:** 1 Department of Oral and Maxillofacial Surgery, Pacific Dental College and Hospital, Udaipur, IND; 2 Department of Oral and Maxillofacial Surgery, Army College of Dental Sciences, Secunderabad, IND

**Keywords:** salivary gland, sialolith, dental, surgical, submandibular, stone, excision

## Abstract

Salivary stones are termed as sialoliths, and the condition is referred to as sialolithiasis. Pain and swelling in the affected area, especially after eating, occur often. Small, easily accessible stones may be managed with conservative methods, such as milking of ducts, along with palliative care, whereas bigger, more difficult-to-reach stones need surgical removal. In this article, we describe a case of sialolithiasis affecting the right submandibular salivary gland, which was treated by removing the gland and stone surgically. When big stones and the gland are removed extra orally, the results are favorable. Submandibular gland sialoliths are the most frequent kind of salivary gland illness. The treatment of this salivary system problem depends on the patient's clinical history, the size of the sialolith, and the degree of cooperation.

## Introduction

The submandibular salivary gland is a large, mixed mucous gland that is composed of two lobes, a larger surface lobe and a smaller deep lobe, that join together at the posterior border of the mylohyoid muscle [1]. Calcified structures called sialoliths may be seen in the salivary glands' parenchyma or ductal system [2]. One of the most prevalent illnesses affecting the salivary glands is sialolithiasis. It accounts for 1.2% of all cases with enlarged salivary glands on one side of the mouth. The submandibular gland is the typical location for salivary stones (84%) [3]. This article details a clinical instance of right-sided mandibular sialolithiasis that was successfully treated by the removal of the submandibular salivary gland.

## Case presentation

A 56-year-old woman came to see us because she had experienced swelling and intermittent discomfort on the right side of her jaw for the previous six months. She reported swelling that initially was asymptomatic and later was associated with pain during meal times only and occasional fever and malaise. Dental problems in the past or in the family did not matter. Normal ranges were found in routine blood tests. The most recent attack occurred one month before the scheduled appointment, and the discomfort has persisted ever since. No blood or discharge accompanied the edema.

**Figure 1:**
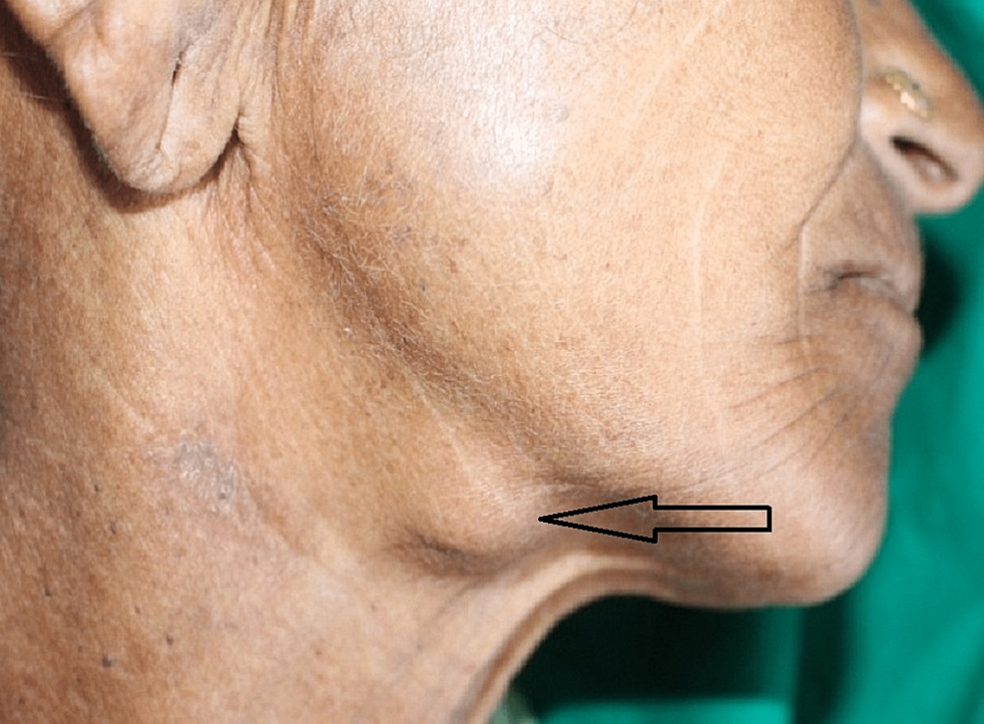
Extraoral swelling

An extraoral examination revealed a firm-to-hard, non-tender, 20x10 mm swelling that extended anteriorly from the right body region to the right angle of the mandible and posteriorly and superiorly from the right inferior border of the mandible to inferiorly, roughly at the level of the hyoid bone (Figure 1).

**Figure 2:**
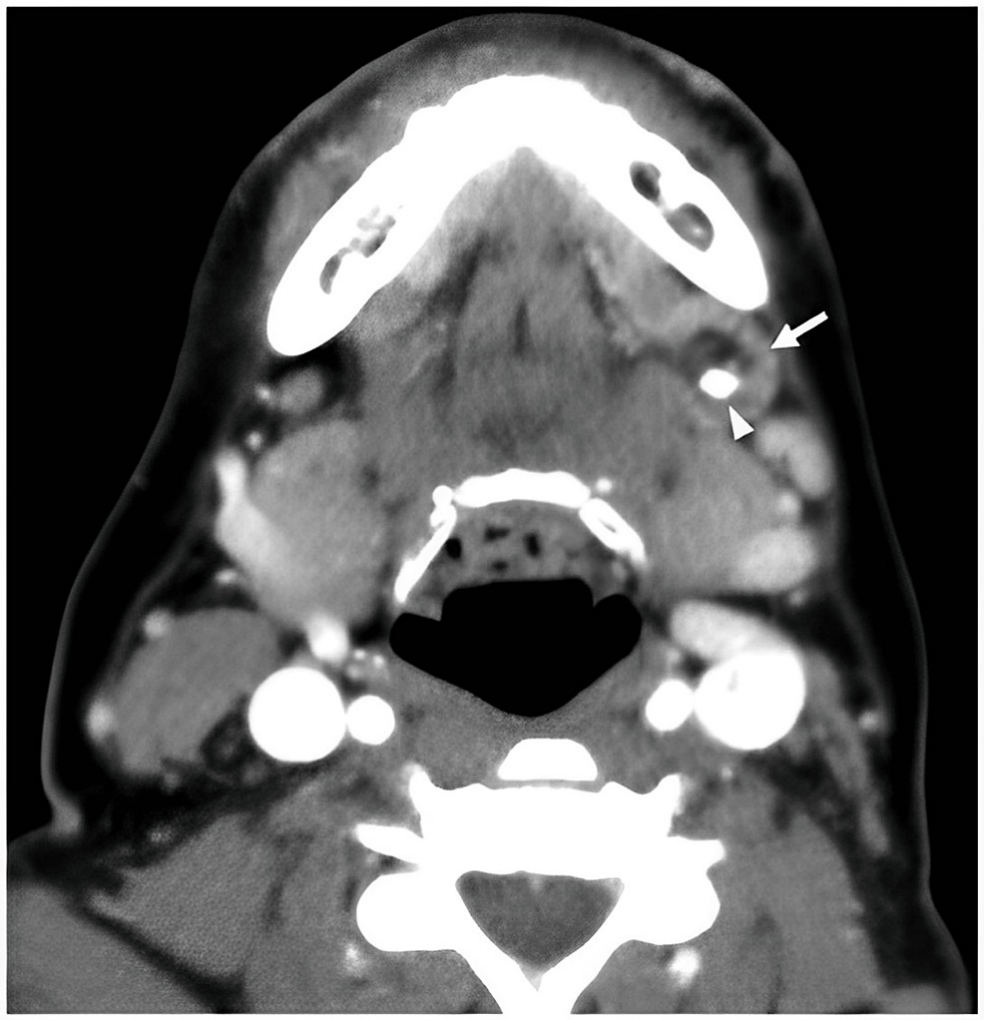
CT scan neck showing the sialolith

There was no facial asymmetry. The patient's mouth opening was adequate. The right submandibular gland was hard, and there was just one sensitive lymph node in that area. On intraoral examination, swelling was present in the right submandibular region, opposite 44 and 46. The right submandibular duct opening was inflamed, erythematous, and edematous. We arrived at a clinical diagnosis of right submandibular sialoadenitis as a result of a sialolith. Computed tomography (CT) was advised, which confirmed our diagnosis (Figure 2).

Accordingly, the excision of the sialolith with the right submandibular salivary gland was the treatment plan decided under general anesthesia. All preoperative investigations were advised and found to be normal.

**Figure 3:**
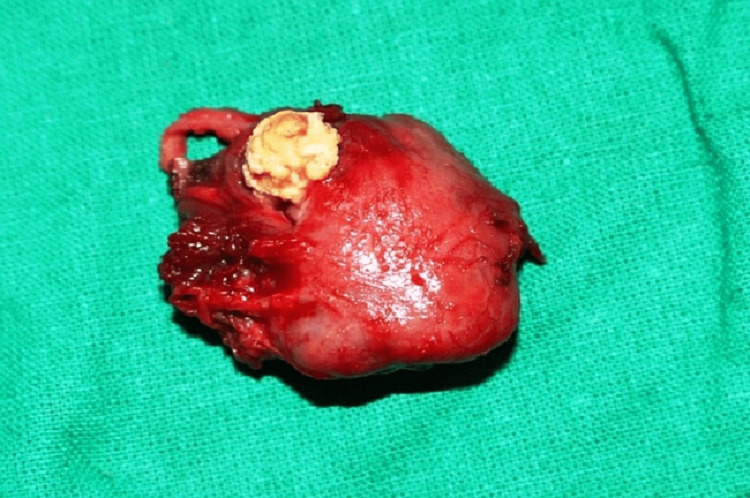
Sialolith along with the submandibular gland

Electrocautery was used to create an extraoral risdon incision beginning at the right angle of the jaw and finishing in the right parasymphysis area (3-4 cm) of the mandible to access the lesion. The subcutaneous tissue, platysma, and submandibular gland capsule were all revealed after the incision was made through the skin. One of the face veins was tied off. The submandibular gland was dissected via its capsule. The marginal mandibular nerve was protected. The calculi, which were spherical in shape and approximately 20x20 mm in size, were removed along with the submandibular gland (Figure 3).

**Figure 4:**
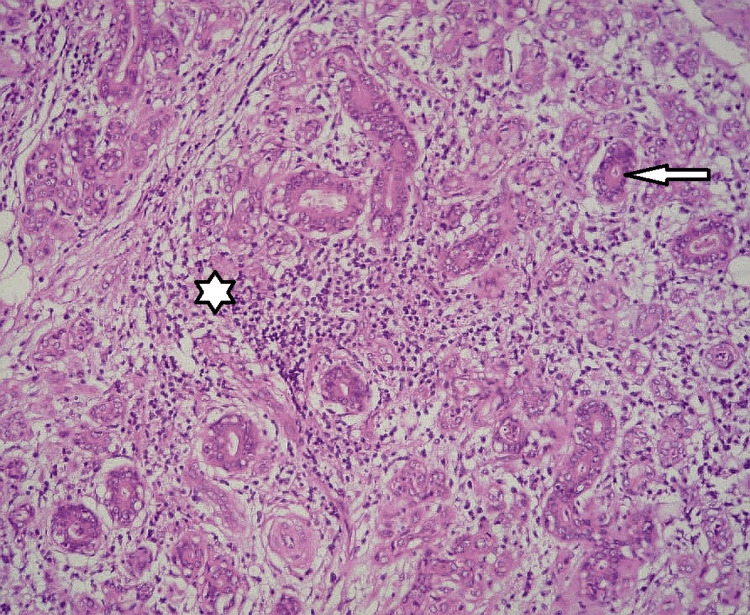
Histopathological section *: dense inflammatory infiltrate; arrow head: acini cells

The wound was irrigated and closed in layers with 3-0 Vicryl to platysma and subcuticular suture to the skin. A suction drain was placed and left in situ .The patient had not experienced any postoperative complications or recurrence on follow-up visits up to two years. The histopathological analysis revealed a salivary parenchyma with salivary acini and ducts, divided by connective tissue septae. Hematoxylin-eosin staining revealed that the extracted submandibular gland was primarily made up of inflammatory salivary tissue. Around the salivary tissue, lymphocytes and plasmacytes, chronic inflammatory cells, were seen migrating mildly to severely. Adipose tissue developed from some salivary tissue. The salivary duct lengthened due to the inflammatory cells' migration, and the acinic cells atrophied (Figure 4).

## Discussion

The submandibular gland is located in the submandibular triangle, a bony depression produced by the jaw at its base and the anterior and posterior digastric stomachs. The gland's medial surface is where Wharton's duct emerges [1]. Sialoliths are condensations of calcium salts and are the most widespread disorder of the major and minor salivary glands. It occurs with equal predilection in males and females [2]. They are not commonly observed in children [4], as the formation of the stones in children requires a longer time, and the concentration of calcium and phosphate in saliva at rest increases with age [5]. The majority of instances of sialolithiasis (75%) are unilateral, whereas 3% are bilateral and 1.2% are atrophic. Stones are more frequent in the small salivary glands between the ages of 5 and 8, whereas stones in the larger salivary glands are more common between the ages of 5 and 8 across the board.

Most of these formations result from the accumulation of mineral salts surrounding an organic nidus consisting of bacteria, salivary mucins, and desquamated epithelial cells [4].

The reverse hypothesis of sialolithiasis suggests that oral bacteria or chemicals might travel into the salivary duct and cause calcification there [6]. Reduced saliva production, dehydration, and a change in salivary pH may result from a number of factors, including impaired crystalloid solubility, excessive alkalinity, increased calcium content, physical injury to the salivary duct or gland, and oropharyngeal infection. It has been shown that smoking, diuretic usage, and systemic disorders like gout all have a role in the development of calculi [7,8]. The definite cause in our case remains unspecified.

Salivary stones are the condensation of organic components, including bacteria or desquamated cells, glycoproteins, collagen, lipids, other proteins, and carbohydrates, along which inorganic or mineral salts in the form of hydroxyapatite, whitlockite, and brushite have precipitated [8]. Approximately 84% of sialoliths are found in the submandibular gland, followed by the parotid gland (13%), sublingual gland (6%), and minor salivary glands (6%). Most stones in the submandibular gland are located in the duct (34% in the distal duct compared to 57% in the hilum). Only 10% of sialoliths are located in the lower lip, whereas 47% are found in the upper lip, 35% in the buccal mucosa, and 10% elsewhere in the minor salivary glands. The left submandibular gland is more likely to develop a sialolith than the right [7], which is contrary to our case. Submandibular glands are most frequently involved, owing to factors, such as antigravity flow of saliva, increased calcium and mucin content of saliva produced by the gland, alkaline nature of the saliva, and a tortuous and longer course of the duct [4].

The patient typically presents with intermittent swelling and pain during meals on the involved side, followed by episodes of remission [8]. Similar features were also present in our patient. Pain is associated with trismus, lymphadenopathy, and pus discharge, along with systemic infections. According to Siddique, some cases can be asymptomatic, irrespective of the size of the stone [4]. The differential diagnoses are phleboliths, abscessed teeth, cervical lymphadenopathy, calcified lymph nodes, tonsillitis, calcified hemangiomas, and benign salivary gland tumours [1,8]. A complete history, clinical and physical examination, and proper imaging modalities help in establishing the right diagnosis and treatment plan. There are a wide variety of radiographic studies that may be performed, including traditional radiography, sialography, ultrasonography, CT, magnetic resonance imaging (MRI), scintigraphy, and sialoendoscopy. In situations when the presence of calculus is in the distal region of the duct and the injected contrast medium impedes the clearance by shifting the calculus toward the gland, sialography is contraindicated [1]. Scintigraphy is advised in such cases. X-rays do not detect all submandibular lithiasis because most of them are radiopaque [8]. CT scans, being noninvasive, are the modality of choice for detecting sialoliths. According to Pachisia et al., the fact that smaller calculi (>2 mm) remain undetected is a major drawback of ultrasonography [3]. Sialo endoscopy is minimally invasive and provides a good visualization of the ductal system, which is advised in cases when CT is unable to detect calculus [8].

The strategy for treating sialolithiasis changes depending on the sialolith's location and size. When the stone is smaller in size, non-invasive conservative management of sialolithiasis is preferred, which comprises gland massage after each meal, use of sialagogues, irrigation, and intake of lots of water. Many new-age treatments have come up, including endoscopic laser lithotripsy, which combines sialendoscopy with laser energy to fragment salivary stones. The laser is delivered through the endoscope, targeting the stone to break it into smaller fragments. This method allows for precise stone fragmentation while minimizing damage to the surrounding tissues. It has shown good results for treating larger or hard-to-reach stones. Salivary duct stenting involves the placement of a small tube or stent in the duct system to keep it open and allow the stone to pass or facilitate future interventions. Stenting can be performed during sialendoscopy or as a separate procedure. It is particularly useful for cases with strictures or recurrent sialolithiasis. Extracorporeal shock wave lithotripsy (ESWL) is a non-invasive technique that uses high-energy shock waves to fragment salivary stones into smaller pieces. The fragmented stones can then pass through the duct system more easily. This method has shown promising results for stones located in the parotid gland and larger submandibular stones.

An intraoral approach under local anaesthesia is the treatment choice for the removal of most of the intraductal submandibular and parotid stones [2]. In cases of large stones, invasive techniques, such as extracorporeal shock-wave lithotripsy, endoscopic intracorporeal shock-wave lithotripsy, sialo endoscopy, CO_2_ laser-guided removal, surgical removal under general anaesthesia, or a combination of approaches are the treatment modalities of choice [3,9]. Considering the chief complaint, clinical features, and CT findings, we decided to remove the sialolith along with the gland under general anesthesia (GA). Complications associated with surgical approaches include salivary fistula, neurological damage, facial scarring, and Frey’s syndrome [3]. Recurrence is found in 1-10% of patients [2]. Our patient has not experienced any postoperative complications or recurrences on follow-up visits.

## Conclusions

Sialolithiasis is a common disorder of the salivary gland. Preoperative history and clinical and radiological examinations are beneficial to arrive at the final diagnosis and treatment plan. Excision of the gland should be indicated following failure of other treatment modalities and in cases with stones of large sizes located in the inaccessible area of the gland.
